# Supplementary material for: The central role of arginine in Haemophilus influenzae survival in a polymicrobial environment with Streptococcus pneumoniae and Moraxella catarrhalis
Source: PLoS One. 2022 Jul 25;17(7):e0271912. doi: 10.1371/journal.pone.0271912 (PMC9312370; doi:10.1371/journal.pone.0271912)
Supplement: S1 Fig — Differential gene expression of artM in H. influenzae 86-028NP (86), H. influenzae in co-culture with S. pneumoniae (86+11), H. influenzae in co-culture with M. catarrhalis (86+QC), and H. influenzae in triple-species culture (Triple). Gene expression in presented as % gene expression relative to artM expression in 86-028NP in mono-culture. The data represents the average of 2 independent experiments, each performed in triplicate (*, p<0.1). (DOCX) [file pone.0271912.s001.docx]

**S1 Fig.** Differential gene expression of *artM* in *H. influenzae* 86-028NP (86), *H. influenzae* in co-culture with *S. pneumoniae* (86+11), *H. influenzae* in co-culture with *M. catarrhalis* (86+QC), and *H. influenzae* in triple-species culture (Triple). Gene expression in presented as % gene expression relative to *artM* expression in 86-028NP in mono-culture. The data represents the average of 2 independent experiments, each performed in triplicate (*, p<0.05).
