# Supplementary material for: The central role of arginine in Haemophilus influenzae survival in a polymicrobial environment with Streptococcus pneumoniae and Moraxella catarrhalis
Source: PLoS One. 2022 Jul 25;17(7):e0271912. doi: 10.1371/journal.pone.0271912 (PMC9312370; doi:10.1371/journal.pone.0271912)
Supplement: S2 Fig — Planktonic growth of mono-, dual- and triple-species cultures of H. influenzae 86-028NP, S. pneumoniae 11 and M. catarrhalis QC at pH 8 over 18h, with (grey) and without (black) supplementation of exogenous arginine at a concentration of 4g/L. Growth is shown for H. influenzae 86-028NP in mono-culture (A), S. pneumoniae 11 in mono-culture (B), M. catarrhalis QC in mono-culture (C), dual-species culture of H. influenzae 86-028NP and S. pneumoniae 11 (D), dual-species culture of H. influenzae 86-028NP and M. catarrhalis QC (E), dual-species culture of S. pneumoniae 11 and M. catarrhalis QC (F), and triple-species culture (G). (DOCX) [file pone.0271912.s002.docx]

**S2 Fig.** Planktonic growth of mono-, dual- and triple-species cultures of *H. influenzae* 86-028NP, *S. pneumoniae* 11 and *M. catarrhalis* QC at pH 8 over 18h, with (grey) and without (black) supplementation of exogenous arginine at a concentration of 4g/L. Growth is shown for *H. influenzae* 86-028NP in mono-culture (A), *S. pneumoniae* 11 in mono-culture (B), *M. catarrhalis* QC in mono-culture (C), dual-species culture of *H. influenzae* 86-028NP and *S. pneumoniae* 11 (D), dual-species culture of *H. influenzae* 86-028NP and *M. catarrhalis* QC (E), dual-species culture of *S. pneumoniae* 11 and *M. catarrhalis* QC (F), and triple-species culture (G).
