# Supplementary material for: The central role of arginine in Haemophilus influenzae survival in a polymicrobial environment with Streptococcus pneumoniae and Moraxella catarrhalis
Source: PLoS One. 2022 Jul 25;17(7):e0271912. doi: 10.1371/journal.pone.0271912 (PMC9312370; doi:10.1371/journal.pone.0271912)
Supplement: S3 Fig — Intracellular pH of H. influenzae following 30min incubation in the presence of 0, 2 or 4g/L of supplemented arginine. (DOCX) [file pone.0271912.s003.docx]

**S3 Fig.** Intracellular pH of *H. influenzae* following 30min incubation in the presence of 0, 2 or 4g/L of supplemented arginine.
