# Supplementary material for: The central role of arginine in Haemophilus influenzae survival in a polymicrobial environment with Streptococcus pneumoniae and Moraxella catarrhalis
Source: PLoS One. 2022 Jul 25;17(7):e0271912. doi: 10.1371/journal.pone.0271912 (PMC9312370; doi:10.1371/journal.pone.0271912)
Supplement: S1 Table — (DOCX) [file pone.0271912.s004.docx]

| **S1 Table. Genes up-regulated in *H. influenzae* 86-028NP following 2 h growth in co-culture with *S. pneumoniae* 11** | | | |
| --- | --- | --- | --- |
| Up-regulated Gene ID | Fold Change | pval (<0.01) | Gene |
| **Gene class: Arginine transport** |  |  |  |
| NTHI_RS06370 | 2.11 | 6.49E-06 | arginine ABC transporter substrate-binding *artI* |
| **Gene class: Amino sugar and sialic acid metabolism** |  |  |  |
| NTHI_RS01125 | 2.11 | 8.63E-14 | N-acetylneuraminate lyase |
| NTHI_RS03135 | 2.13 | 0.001692 | aspartate ammonia-lyase |
| **Stress response** |  |  |  |
| NTHI_RS03345 | 2.14 | 3.35E-09 | hybrid peroxiredoxin hyPrx5 |
