# Supplementary material for: The central role of arginine in Haemophilus influenzae survival in a polymicrobial environment with Streptococcus pneumoniae and Moraxella catarrhalis
Source: PLoS One. 2022 Jul 25;17(7):e0271912. doi: 10.1371/journal.pone.0271912 (PMC9312370; doi:10.1371/journal.pone.0271912)
Supplement: S2 Table — (DOCX) [file pone.0271912.s005.docx]

| **S2 Table. Genes up-regulated in *H. influenzae* 86-028NP following 2 h growth in triple species co-culture with *S. pneumoniae* 11 *and M. catarrhalis* QC** | | | |
| --- | --- | --- | --- |
| **Up-regulated Gene ID** | Fold Change | pval (<0.01) | Gene |
| **Gene class: Stress response** |  |  |  |
| NTHI_RS07935 | 2.15 | 0.00012 | cold-shock protein *cspD* |
| **Gene class: Arginine uptake** |  |  |  |
| NTHI_RS06370 | 2.29 | 0.005212 | arginine ABC transporter substrate-binding *artI* |
| NTHI_RS06375 | 3.18 | 0.009696 | arginine ABC transporter ATP-binding protein *artP* |
| **Gene class: Amino sugar and sialic acid metabolism** |  |  |  |
| NTHI_RS01120 | 2.18 | 5.92E-08 | glucosamine-6-phosphate deaminase |
| NTHI_RS01125 | 2.21 | 2.74E-19 | N-acetylneuraminate lyase |
| NTHI_RS01140 | 2.92 | 4.18E-05 | N-acetylmannosamine-6-phosphate 2-epimerase |
| NTHI_RS01135 | 2.26 | 0.000242 | N-acetylmannosamine kinase |
| **Ribose degradation** |  |  |  |
| NTHI_RS02980 | 2.05 | 9.58E-05 | D-ribose pyranase |
| **Up-regulated genes specific to the triple co-culture condition** |  |  |  |
| NTHI_RS02950 | 2.01 | 0.001801 | acid phosphatase/phosphotransferase |
| **Gene class: Acid tolerance** |  |  |  |
| NTHI_RS03135 | 2.08 | 8.38E-05 | aspartate ammonia-lyase |
| NTHI_RS00700 | 2.14 | 1.27E-10 | hypothetical protein |
