# Supplementary material for: The central role of arginine in Haemophilus influenzae survival in a polymicrobial environment with Streptococcus pneumoniae and Moraxella catarrhalis
Source: PLoS One. 2022 Jul 25;17(7):e0271912. doi: 10.1371/journal.pone.0271912 (PMC9312370; doi:10.1371/journal.pone.0271912)
Supplement: S3 Table — (DOCX) [file pone.0271912.s006.docx]

| **S3 Table. Genes down-regulated in *H. influenzae* 86-028NP following 2 h co-culture with *S. pneumoniae* 11** | | | |
| --- | --- | --- | --- |
| Down-regulated Gene ID | Fold Change | pval (((<valval | Gene |
| NTHI_RS02555 | -2.42 | 2.20E-21 | thiamine metabolism 4-methyl-5-beta-hydroxyethylthiazole kinase |
| NTHI_RS02560 | -2.32 | 3.60E-19 | hydroxymethylpyrimidine  kina kinase |
| NTHI_RS02565 | -2.32 | 1.30E-18 | TMP pyrophosphorylase |
| NTHI_RS02570 | -2 | 1.67E-14 | metabolite transport protein |
