# Supplementary material for: The central role of arginine in Haemophilus influenzae survival in a polymicrobial environment with Streptococcus pneumoniae and Moraxella catarrhalis
Source: PLoS One. 2022 Jul 25;17(7):e0271912. doi: 10.1371/journal.pone.0271912 (PMC9312370; doi:10.1371/journal.pone.0271912)
Supplement: S4 Table — (DOCX) [file pone.0271912.s007.docx]

| **S4 Table. Genes down-regulated in *H. influenzae* 86 following 2 h growth in triple-species co-culture with *S. pneumoniae* 11 and *M. catarrhalis* QC** | | | |
| --- | --- | --- | --- |
| Downregulated genes ID | Fold Change | pval (<0.01) | Gene |
| NTHI_RS02560 | -2.11 | 2.33E-21 | bifunctional hydroxymethylpyrimidine kinase |
| NTHI_RS02255 | -2.05 | 1.45E-07 | ABC transporter permease |
| NTHI_RS02555 | -2.02 | 1.40E-19 | hydroxyethylthiazole kinase |
